# *EVA1B* to Evaluate the Tumor Immune Microenvironment and Clinical Prognosis in Glioma

**DOI:** 10.3389/fimmu.2021.648416

**Published:** 2021-04-06

**Authors:** Shanqiang Qu, Jin Liu, Huafu Wang

**Affiliations:** ^1^ Department of Neurosurgery, Nanfang Hospital, Southern Medical University, Guangzhou, China; ^2^ Department of Neurosurgery, Lishui People’s Hospital (The Sixth Affiliated Hospital of Wenzhou Medical University), Lishui, China; ^3^ Department of Clinical Pharmacy, Lishui People’s Hospital (The Sixth Affiliated Hospital of Wenzhou Medical University), Lishui, China

**Keywords:** *EVA1B*, prognosis, glioma, biomarker, microenvironment

## Abstract

**Background:**

Previous research indicated that the tumor cells and microenvironment interactions are critical for the immunotherapeutic response. However, predicting the clinical response to immunotherapy remains a dilemma for clinicians. Hence, this study aimed to investigate the associations between *EVA1B* expression and prognosis and tumor-infiltrating immune cells in glioma.

**Methods:**

Firstly, we detected the *EVA1B* expression in glioma tissues through biological databases. The chi-squared test, Kaplan-Meier, and univariate and multivariate Cox regression analyses were used to analyze the clinical significance of *EVA1B* expression. The correlation between *EVA1B* expression and levels of tumor-infiltrating immune cells in glioma tissues was investigated. Receiver operating characteristic (ROC) analysis was performed to compare the predictive power between *EVA1B* and other commonly immune-related markers.

**Results:**

In the CGGA cohort of 325 glioma patients, we found that *EVA1B* was upregulated in glioma, and increased with tumor grade. High *EVA1B* expression was prominently associated with unfavorable clinicopathological features, and poorer survival of patients, which were further confirmed by TCGA (n=609) and GEO (n=74) cohorts. Furthermore, multivariate analysis indicated that *EVA1B* is an independent prognostic biomarker for glioma. Importantly, *EVA1B* overexpression was associated with a higher infiltration level of CD4^+^ T cells, CD8^+^ T cells, B cells, macrophages, and neutrophils in glioma. ROC curves showed that, compared with *PD-L1*, *CTLA-4*, and *Siglec15*, *EVA1B* presented a higher area under the curve (AUC) value (AUC=0.824) for predicting high immune infiltration levels in glioma.

**Conclusions:**

We found that *EVA1B* was upregulated and could act as a poor prognostic biomarker in glioma. Importantly, *EVA1B* overexpression was associated with the immune infiltration levels of immune cells including B cells, CD4^+^ T cells, CD8^+^ T cells, macrophages, and neutrophils, and strongly with the overall immune infiltration levels of glioma. These findings suggested that *EVA1B* might be a potential biomarker for evaluating prognosis and immune infiltration in glioma.

## Introduction

Glioma is one of the brain malignancies with the highest prevalence, and the incidence of glioma is on the increase worldwide (1, 2). Despite the variety of modern therapies against glioblastoma, it is still a deadly disease with an extremely poor prognosis. The median overall survival (OS) of glioblastoma (GBM) patients is less than two years (3). Most patients have a few months of survival if they are left untreated (4). Therefore, it is of great significance to indicate how to develop further effective therapeutic strategies to improve the OS of glioma patients.

In recent years, immunotherapy has increasingly attracted scholars’ attention in the field of tumor therapy worldwide (5). Since January 2015, cancer immunotherapy has made significant advances (6). The programmed cell death protein 1 (*PD-1*) blockade is effective in the treatment of Hodgkin’s lymphoma (HL) and raises hopes that it may change the treatment pattern of the disease (7). Immunotherapy, such as cytotoxic T lymphocyte associated antigen 4 (*CTLA4*), programmed death-1 (*PD-1*), and programmed death ligand-1 (*PD-L1*) inhibitors, showed promising antitumor effects in malignant melanoma (8). However, current immunotherapies, such as anti-CTLA4, anti-PD-1, and anti-PD-L1, showed poor clinical efficacy in GBM (9). The reason for the ineffectiveness of the treatment is very complicated, which may be related to the lack of biomarkers for guiding individualized immune targets (9). Increasing studies have also found that tumor-infiltrating lymphocytes, such as tumor associated macrophages (TAMs) and tumor-infiltrating neutrophils (TINs), affect the prognosis and efficacy of chemotherapy and immunotherapy (10). Thus, there is an urgent need for the elucidation of the immunophenotypes of tumor-immune interaction and identification of novel immune-related biomarkers and therapeutic targets in glioma.

In humans, the *EVA1* family includes three main groups: *EVA1A*, *EVA1B*, and *EVA1C*. Previous studies have revealed that *EVA1A*, an endoplasmic reticulum-related protein were involved in regulating autophagy and apoptosis-related mechanisms in many cancers (11, 12). Additionally, Bang-Yi Lin et al. found that *EVA1A* promotes papillary thyroid cancer progression and epithelial-mesenchymal transition *via* upregulating immune-related signaling pathways (11). Hippo signaling pathway. *MST1/2* is the core kinases of the classical Hippo signaling pathway in mammals (13), which also plays a key role in the immune microenvironment by regulating the adhesion and trafficking of immune cells (14). A paralogous gene of the *EVA1A* gene is *EVA1B*, which both belong to the *EVA1* family. *EVA1B* has high sequence similarity with the *EVA1A* gene. We also found that the *EVA1B* protein has the same domain as the *EVA1A* protein using the GenesLikeMe database. However, what role *EVA1B* plays in tumors has not yet been reported, especially its correlation with immune infiltration.

Consequently, this study was conducted to comprehensively identify *EVA1B* expression levels in glioma, and its correlation with malignant features and the prognosis of glioma patients. Moreover, we also investigated the correlation of *EVA1B* with infiltration levels of different immune cells and further assessed the predictive performance of *EVA1B* for high infiltration levels of glioma.

## Materials and Methods

### Gene Expression Profiles

The mRNA expression profiles of EVA1B were obtained from the GEO (https://www.ncbi.nlm.nih.gov/geo/) and TCGA database (http://cancergenome.nih.gov/). Moreover, a pan-cancer analysis of *EVA1B* was conducted using the Oncomine (http://www.oncomine.org) and the online tool TIMER (https://cistrome.shinyapps.io/timer/). Additionally, the localization of EVA1B protein was analyzed by immunofluorescence images from the Human Protein Atlas (HPA) database (https://www.proteinatlas.org/).

### Patients

The included glioma patients were retrospectively collected from the CGGA database (http://cgga.org.cn/, Dataset ID: mRNAseq_325) and acted as a CGGA cohort. A total of 325 glioma patients were analyzed, and general clinical data and RNA-sequencing data were retrieved from the CGGA database. Among them, the mean age of included patients was 42.94 years (age range: 8-79 years). The details of patients’ characteristics are summarized in **Supplementary Table 1**. Ethical approval was obtained through the Ethics Committee of Lishui People’s Hospital, written informed consent was waived by the ethics committee because all data comes from public databases.

### Validation of External Datasets (TCGA and GEO Databases)

To validate the prognostic value of *EVA1B* in glioma, two independent datasets from TCGA and GEO databases were used to validate the TCGA (n=609) and GEO (n=74) cohorts. In the TCGA cohort, 609 glioma patients (mean age=47.3 years, range: 14-89 years) were included. The details of patients’ characteristics are summarized in **Supplementary Table 1**. In the GEO cohort, 74 glioma patients (age range, 18-82) were included, and 32 (43.2%) of them were male and 42 (56.8%) were female. The correlation between *EVA1B* expression and prognosis of patients was analyzed by the comprehensive platform PrognoScan website (http://www.prognoscan.org/).

### Biological Functions of *EVA1B* Participation in Glioma

First of all, the top 500 genes most co-expressed with *EVA1B* were extracted from the LinkedOmics database (http://www.linkedomics.org) (15). Function annotations were conducted to identify the biological processes potentially involved, cellular components, molecular function, and signaling pathway using DAVID 6.8 (https://david.ncifcrf.gov/) (16). To identify the potential interacting proteins, a functional protein network was built by GeneMANIA software (http://www.genemania.org/) (17).

### Systematic Analysis of Immune Infiltration

To comprehensively investigate the associations between *EVA1B* and immune cells and immune molecules, we used the TIMER and EPIC algorithm to estimate the abundance of six immune infiltrates including B cells, CD4^+^ T cells, CD8^+^ T cells, Neutrophils, Macrophages, and Dendritic cells (DCs) (18).

### Statistical Analysis

In this study, statistical analysis was performed using SPSS (Version 23.0; IBM), GraphPad (version 6.0), and R software (version 3.6.1). Chi-square test was used to calculate categorical variables. For continuous variables, when continuous variables follow the normal distribution, an independent Student’s t-test was conducted. Otherwise, a Mann-Whitney test was conducted. Spearman rank tests were also performed for correlation analysis. Kaplan-Meier analysis was performed to compare the OS of patients. Univariate and multivariate analyses were used to explore the prognostic factor for glioma patients. A time-dependent ROC curve was performed to evaluate prediction power. All *P*-value <0.05 was considered statistically significant (*P<0.05, **P<0.01, ***P<0.001, ****P<0.0001).

## Results

### Expression of *EVA1B* in Various Tumor Types

**Figure 1:**
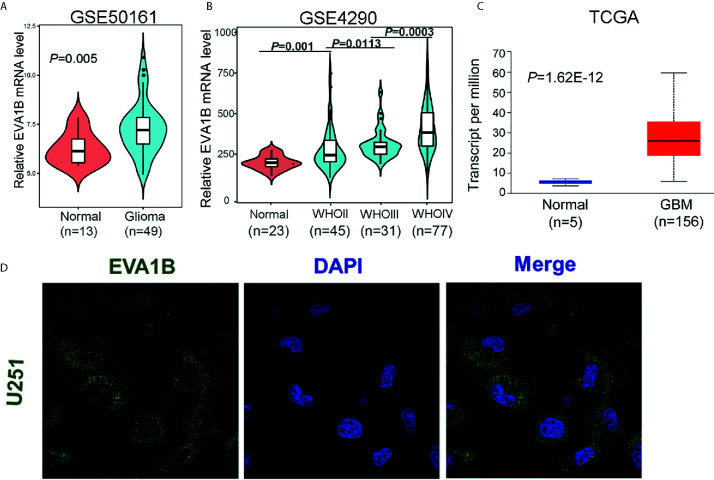
The expression of *EVA1B* is highly expressed in glioma compared to the normal brain tissues. **(A–C)** The *EVA1B* expression is significantly regulated in glioma in the GSE50161, GSE4290, and TCGA, and increases with tumor grade. **(D)** The *EVA1B* protein is predominantly localized in the cytoplasm and cellular membrane of U251 (images obtained from the Human Protein Atlas).

Firstly, we compared the differential mRNA level of *EVA1B* between the normal brain tissue and glioma tissues from the GEO and TCGA database. Compared with normal tissues, *EVA1B* mRNA expression was remarkably upregulated in gliomas based on GSE50161 (normal: 13, glioma: 49, **Figure 1A**) and GSE4290 (normal: 23, WHOII: 45, WHOIII: 31, WHOIV: 77, **Figure 1B**), and the *EVA1B* expression level increased with tumor grade. Similar results were obtained by analyzing TCGA sequencing data of glioma tissues (normal: 5, GBM: 156, **Figure 1C**). To gain insight into the subcellular localization of *EVA1B* protein, we obtained the immunofluorescence images of *EVA1B* in the U251 cell line from the HPA database, which contains high-resolution, multi-color immunofluorescence images of cells that detail the subcellular distribution pattern of proteins. Immunofluorescence analysis showed that *EVA1B* protein mainly localized to cytoplasm and membrane (**Figure 1D**). In total, the *EVA1B* expression levels in glioma were significantly overexpressed and were increased with tumor grade.

We also explored the *EVA1B* expression level in pan-cancer. As expected, compared with normal tissue, the *EVA1B* expression was also upregulated in breast cancer, colorectal cancer, lymphoma, melanoma, and sarcoma based on the Oncomine database (**Supplementary Figure 1A**). To confirm these findings, we further analyzed the TCGA data of tumors and obtained similar results (**Supplementary Figure 2B**).

### The Association Between *EVA1B* Expression and the Clinicopathological Features of Glioma Patients

**Figure 2:**
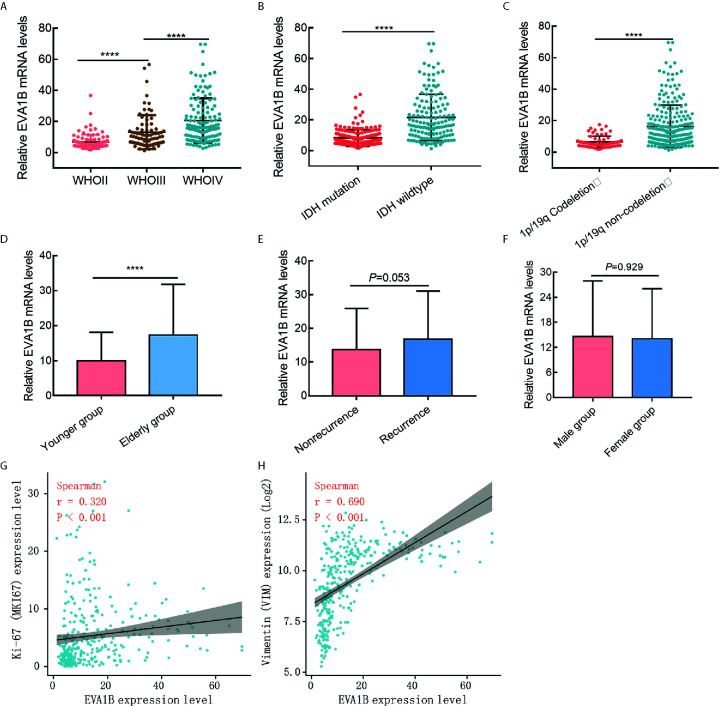
The association between *EVA1B* expression and clinicopathological features in glioma cohort (CGGA cohort, n = 325). **(A–F)** The *EVA1B* expression was significantly associated with WHO grade, IDH wildtype, 1P/19q non-codeletion, and age, but not with recurrence of glioma and sex. **(G, H)** *EVA1B* expression was weakly associated with proliferation marker (Ki-67 expression), and strongly associated with invasion marker (Vimentin expression). (****P < 0.0001).

**Figure 3:**
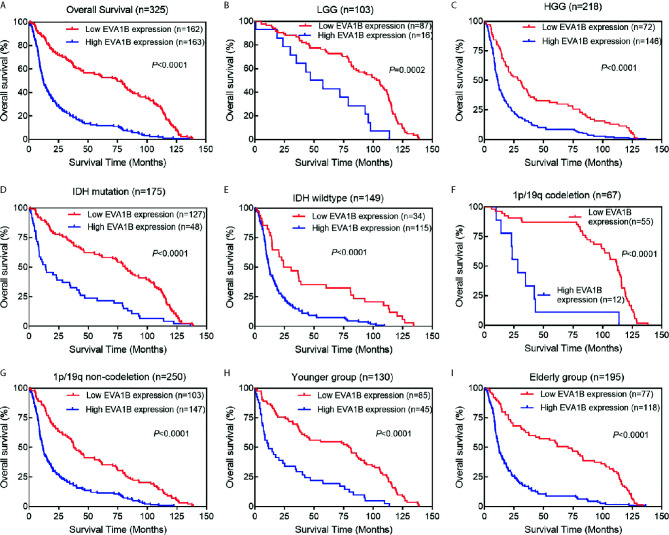
The association between *EVA1B* expression and prognosis of glioma patients. **(A)** Overall, Kaplan-Meier curves showed that patients with *EVA1B* overexpression have a poorer prognosis than those with low expression. **(B–I)** In the subgroup analysis, patients with *EVA1B* overexpression showed significantly poorer prognosis than those with low expression in the LGG subgroup, HGG subgroup, IDH mutation subgroup, IDH wildtype subgroup, 1p/19q codeletion subgroup, 1p/19q non-codeletion subgroup, younger subgroup, and elderly subgroup.

In the above, we reported that *EVA1B* expression was positively related to tumor grade. To further explore *EVA1B* overexpression and malignant behavior of glioma, 325 patients with glioma were obtained from the CGGA database. First, we compared the differential mRNA level of *EVA1B* in different subgroups stratified by WHO grade, IDH mutation, 1p/19q codeletion, age, recurrence, and sex. As shown in **Figures 2A–F**, *EVA1B* expression was significant different between subgroups stratified by WHO grade (*P*<0.0001, *P*<0.0001, respectively, **Figure 2A**), IDH mutation (*P*<0.0001, **Figure 2B**), 1p/19q codeletion (*P*<0.0001, **Figure 3C**), age (*P*<0.0001, **Figure 3D**), but not in subgroups stratified by recurrence (*P*=0.053, **Figure 2E**), and sex (*P*=0.929, **Figure 2F**).

Second, we analyzed its correlation with *MKI67* (Ki-67 proliferation index) and *VIM* expression level (Vimentin invasion index) and found that *EVA1B* was weakly correlated with Ki-67 (r=0.320, *P*<0.001, **Figure 2G**), but strongly correlated with Vimentin expression (r=0.690, *P*<0.001, **Figure 2H**). This result was also verified by the TCGA cohort of 609 glioma patients (**Supplementary Figures 2A, B**).

Third, according to the median value of *EVA1B* expression, all patients were divided into low or high *EVA1B* groups. Chi-square test results showed that overexpressed *EVA1B* was significantly associated with age (*P*<0.001), WHO grade (*P*<0.001), histopathology (*P*<0.001), IDH status (*P*<0.001), and 1p/19q status (*P*<0.001) (**Table 1**, **Supplementary Table 2**). These results suggested that *EVA1B* overexpression was related to the malignant clinicopathological features of glioma patients.

**Table 1:** The correlation between the expression of *EVA1B* and clinicopathological factors (CGGA).

Features	Case (%)	*EVA1B* expression level		*P*-value
		Low expression	High expression	
**Age**				
≥40	130(40.0)	85	45	<0.001
<40	195(60.0)	77	118	
**Sex**				
Male	203(62.5)	103	100	0.678
Female	122(37.5)	59	63	
**WHO grade**				
WHO II	103(31.7)	87	16	<0.001
WHO III	79(24.3)	39	40	
WHO IV	139(42.8)	33	106	
**Histopathology**				
O	26(8.0)	22	4	<0.001
OA	38(11.7)	34	4	
A	39(12.0)	31	8	
AO	12(3.7)	9	3	
AOA	39(12.0)	19	20	
AA	28(8.6)	11	17	
GBM	139(42.8)	33	106	
**IDH**				
Mutation	175(53.8)	127	48	<0.001
Wildtype	149(45.8)	34	115	
**1p/19q**				
Codel	67(20.6)	55	12	<0.001
Non-codel	250(76.9)	103	147	
**MGMTp methylation**				
Yes	157(48.3)	85	72	0.109
No	149(45.8)	67	82	
**Radiotherapy**				
Yes	258(79.4)	131	127	0.459
No	51(15.7)	23	28	
**Chemotherapy**				
Yes	178(54.8)	79	99	0.190
No	124(38.2)	72	52	
**Recurrence**				
Yes	62(19.1)	28	34	0.443
No	259(79.7)	131	128	

O, oligodendroglioma; OA, oligoastrocytoma; A, astrocytoma; AO, anaplastic oligodendroglioma; AOA, anaplastic oligoastrocytoma; AA, anaplastic astrocytoma; GBM, glioblastoma; MGMTp, MGMT promoter.

### The Prognostic Value of *EVA1B* Expression in Glioma Patients

To assess whether *EVA1B* could act as a prognosis marker for glioma patients, Kaplan-Meier survival analysis was conducted to compare the OS of patients between high and low *EVA1B* expression groups. Overall, the Kaplan-Meier curve showed that patients from the high *EVA1B* expression group had a remarkably poorer OS (HR=0.38, 95%CI=0.30-0.49, *P*<0.0001, **Figure 3A**). Furthermore, stratified survival analysis was performed to assess the prognostic significance of *EVA1B* in different subgroups of glioma patients stratified by age, WHO grade, IDH status, and 1p/19q status. The results of stratified survival analysis were consistent with the results shown in **Figure 3A** (**Figures 3B–I**). Next, the univariate and multivariate analysis results revealed that *EVA1B* expression was an independent prognostic biomarker for glioma patients (HR= 1.93, 95%CI=1.42-2.62, *P*<0.001, **Table 2**).

**Table 2:** Univariate and multivariate Cox-regression analysis of clinicopathological factors affecting the prognosis of patients.

Features	Univariate analysis			Multivariate analysis		
	HR	95%CI	*P*-value	HR	95%CI	*P*-value
Age	1.34	1.07-1.69	0.012	0.95	0.72-1.25	0.710
Sex	1.03	0.81-1.29	0.830			
WHO grade	2.18	1.88-2.52	<0.001	0.97	0.56-1.68	0.904
Histopathology	1.41	1.32-1.50	<0.001	1.37	1.08-1.74	0.009
IDH	0.44	0.35-0.56	<0.001	1.05	0.76-1.46	0.760
1p/19q	0.37	0.28-0.50	<0.001	0.73	0.50-1.08	0.113
MGMTp methylation	0.86	0.68-1.08	0.193			
Radiotherapy	0.55	0.40-0.76	<0.001	0.73	0.52-1.04	0.084
Chemotherapy	1.39	1.10-1.77	0.006	0.70	0.53-0.93	0.015
Recurrence	0.60	0.45-0.80	<0.001	0.79	0.57-1.08	0.137
EVA1B expression	3.04	2.39-3.87	<0.001	1.93	1.42-2.62	<0.001

HR, hazard ratio; CI, confidence interval; MGMTp, MGMT promoter.

**Figure 4:**
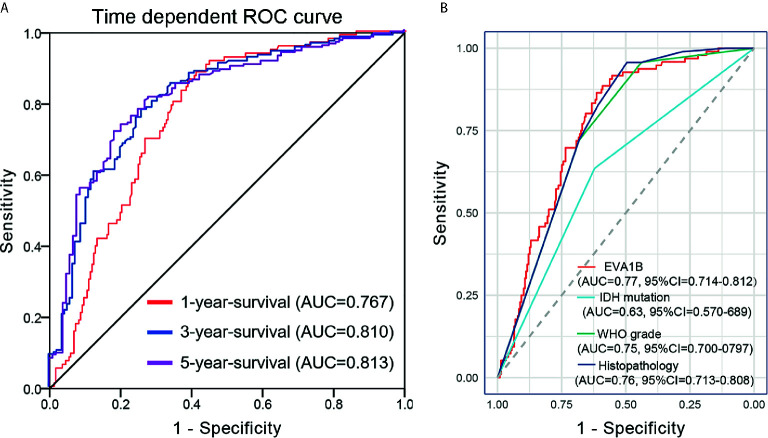
Predictive power for prognosis with *EVA1B* expression by ROC curve analysis. **(A)** ROC curves showed that the AUC of *EVA1B* for predicting 1-, 3- and 5-years survival of patients is 0.767, 0.810, and 0.813, respectively. **(B)** The prediction performance of *EVA1B* protein is higher than IDH mutation, WHO grade, and histopathology.

A time-dependent ROC curve was also used to assess the predictive power of *EVA1B* in predicting 1-, 3- and 5-years OS, and the AUC for 1-, 3- and 5-year survival rate of glioma patients was 0.767, 0.810, and 0.813, respectively (**Figure 4A**). It is noteworthy that, compared with the clinical common indicators including IDH mutation, WHO grade, and histopathology, *EVA1B* had a higher predictive power (**Figure 4B**). Taken together, these results suggest that *EVA1B* is a moderately sensitive index for predicting the prognosis of glioma patients, and can act as an effective prognostic biomarker in glioma.

### Validation of the Prognostic Significance of *EVA1B* in TCGA and GEO Cohorts

To independently verify the prognostic significance of *EVA1B*, data from the TCGA and GEO databases were used as validation cohorts, respectively. In the TCGA cohort of 609 glioma patients, we also compared the differential mRNA level of *EVA1B* in different subgroups based on WHO grade, IDH mutation, and 1p/19q codeletion. As shown in **Supplementary Figures 3A–C**, the results of the TCGA validation cohort are consistent with the results of the CGGA cohort in WHO grade (*P*<0.0001, **Supplementary Figure 3A**), IDH status (*P*<0.0001, **Supplementary Figure 3B**), and 1p/19q status groups (*P*<0.0001, **Supplementary Figure 3C**). Similarly, **Supplementary Table 3** revealed the comparison of clinicopathological features between the high and low *EVA1B* groups, which yielded similar results.

Next, to validate the prognostic value of *EVA1B* for glioma patients, Kaplan-Meier analysis was used to compare the OS between Low-*EVA1B* and high-*EVA1B* groups and showed that patients with high *EVA1B* expression had a remarkably worse OS than patients with low-*EVA1B* expression (*P*<0.0001, **Supplementary Figure 3D**). Likewise, all patients were further stratified by WHO grade and IDH mutation. Kaplan-Meier curves showed that patients with low *EVA1B* were significantly correlated with a much better OS than patients with high *EVA1B* in the LGG subgroup (*P*<0.05, **Supplementary Figure 3E**), HGG subgroup (*P*<0.0001, **Supplementary Figure 3F**), and IDH-wildtype subgroup (*P*=0.0092, **Supplementary Figure 3H**). Although there was no statistical difference between patients with low and high *EVA1B* in the IDH-mutation subgroup (*P*>0.05, **Supplementary Figure 3G**), the median survival of patients with low-*EVA1B* expression was better than patients with high-*EVA1B* expression (105.1 months and 75.1 months, respectively).

We also verified the prognostic value of *EVA1B* for glioma patients by the GEO dataset (GSE4412-GPL96, n=74). The details of GSE4412-GPL96 were summarized in **Supplementary Figure 4A**. Likewise, the 74 glioma patients were divided into low-*EVA1B* and high-*EVA1B* subgroups (**Supplementary Figures 4B, C**). Consistently, patients with high-*EVA1B* expression were associated with shorter OS (*P*=0.001616, **Supplementary Figure 4D**). Taken together, we found that *EVA1B* overexpression was an unfavorable prognostic indicator for glioma patients.

### Gene Function Annotation and Pathway Analysis

**Figure 5:**
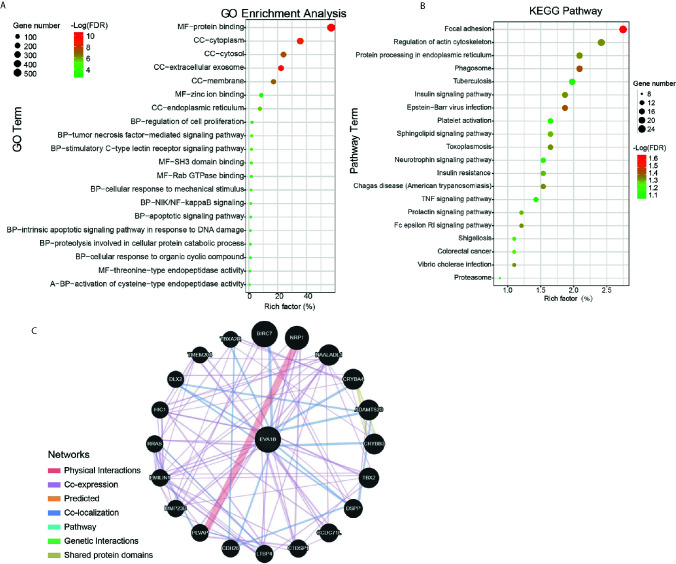
Protein annotation and protein-protein interaction analysis of *EVA1B* gene in glioma. **(A, B)** GO and KEGG pathway analysis of *EVA1B* in glioma. **(C)** Predicted structural proteins essential for the functioning of *EVA1B* generated from GeneMANIA. The different colors of the connecting lines represent the physical interactions, co-expression, predicted interactions, co-localization, the same pathway, genetic interactions, and shared protein domains.

After determining the prognostic value of *EVA1B* in glioma, we next explored what kind of biological function *EVA1B* protein might be associated with. GO and KEGG enrichment analyses were performed, and the top twenty GO terms and signaling pathways are shown in **Figures 5A, B**. *EVA1B* gene was associated with many biological processes such as focal adhesion, regulation of actin cytoskeleton, cell proliferation, and tumor necrosis factor. Of note, *EVA1B* was also associated with NF-kappa B signaling, apoptotic signaling pathway, and TNF signaling pathway. These three signaling pathways are known to be associated with immune and inflammation processes.

To understand the potential interaction proteins of *EVA1*B, a visual interaction network was generated by GeneMANIA software (**Figure 5C**). The GeneMANIA results covered 20 *EVA1B*-interacting proteins, and the proteins are tightly interconnected around *BIRC7, NRP1, NAALADL1, CRYBA4, ADAMTS20, CRYBB3, DSPP, CCDC71L, CTDSP1*, and *LTBP4*.

### Association Between *EVA1B* Expression Levels and Tumor-Immune Microenvironment in Glioma

**Figure 6:**
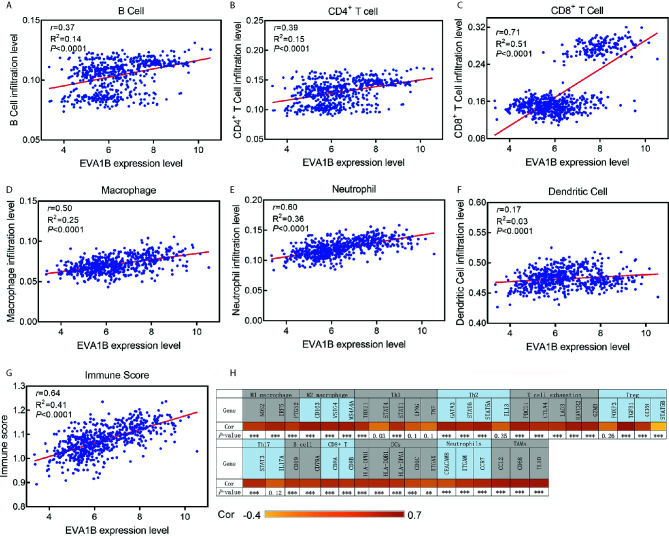
The association between *EVA1B* expression and immune infiltration level. **(A–F)** *EVA1B* expression is correlated with infiltration levels of B cells, CD4^+^ T cells, CD8^+^ T cells, macrophages, neutrophils, but very weakly or not correlated with DCs. **(G)** *EVA1B* expression is strongly correlated with the overall immune score of glioma. **(H)** Heatmap showing the correlation between *EVA1B* expression and the immune markers of various immune cells.

We further explored the correlation between *EVA1B* expression and the infiltration levels of different immune cells in a cohort of 609 glioma patients. We obtained the immune infiltration status of glioma patients by the TIMER algorithm. As shown in **Figures 6A–G**, *EVA1B* expression was shown to be weakly correlated with B cells (*r*=0.37, *P*<0.0001), CD4^+^ T cells (*r*=0.39, *P*<0.0001), moderately with macrophages (*r*=0.50, *P*<0.0001), and also strongly with CD8^+^ T cells (*r*=0.71, *P*<0.0001). However, *EVA1B* was very weakly or not correlated with DCs (*r*=0.17, *P*<0.0001). Importantly, there was a strong correlation between *EVA1B* expression and the overall immune-infiltration levels in glioma (*r*=0.64, *P*<0.0001). These findings suggested that *EVA1B* might be associated with the regulation of immune infiltration of glioma. Next, to verify the correlation between *EVA1B* and immune infiltration levels, we also calculated the infiltration levels of different immune cells by another commonly EPIC algorithm. As expected, we obtained similar results (**Supplementary Figure 5**). In addition, **Figure 6H** shows the correlation between *EVA1B* expression and the various immune marker genes.

Finally, to assess the predictive performance of *EVA1B* for the high infiltration levels of immune cells in glioma, ROC curves were conducted to compare the AUC value between *EVA1B, PD-L1, CTLA-4*, and *Siglec15*. The results revealed that, compared with other markers, *EVA1B* presented a higher predictive power (AUC=0.824, 95%CI=0.79-0.86, *P*<0.0001, **Supplementary Figure 6**).

## Discussion

In this study, we found, for the first time, that *EVA1B* expression was highly upregulated in glioma tissues compared with normal brain tissues, and increased with tumor grade. The pan-cancer analysis also revealed that *EVA1B* was broadly and highly expressed in multiple kinds of tumors. In glioma, we observed that *EVA1B* overexpression was associated with age, WHO grade, histopathology, IDH wildtype, 1p/19q non-codeletion, and marker of invasion, which suggested that *EVA1B* overexpression may be involved in the malignant behavior of glioma. What is more, multivariate Cox regression analysis showed that *EVA1B* overexpression is an independent poor factor for the prognosis of glioma patients, which was also confirmed in two other independent cohorts. Functional enrichment analysis (DAVID) also indicated that *EVA1B* overexpression was associated with tumor proliferation, focal adhesion, regulation of actin cytoskeleton, anti-apoptotic effect, and the inflammatory response, which was in agreement with the above findings. These results implied that *EVA1B* overexpression may have played a key role in the malignant phenotypes of gliomas, and is a potentially unfavorable prognostic biomarker for glioma patients. However, the role *EVA1B* plays in the tumor-immune microenvironment is not reported.

The *EVA1* family protein has three members, *EVA1A, EVA1B*, and *EVA1C*. To date, functional studies of *EVA1B* protein in glioma are still not reported. Lan Wang et al. first reported that *EVA1A* is a novel transmembrane protein (19), and a highly homologous protein of *EVA1B*. Recently, Bang-Yi Lin et al. further found that, compared with the normal tissues, the *EVA1A* was overexpressed in thyroid cancer tissues, and overexpression of *EVA1A* significantly promoted cell proliferation, metastasis, and apoptosis through the Hippo signaling pathway (20). In this study, we observed that *EVA1B* is associated with many biological processes such as focal adhesion, regulation of actin cytoskeleton, cell proliferation, and tumor necrosis factor. Focal adhesion mediates cell migration, survival, proliferation, and apoptosis, which are regulated by tyrosine kinase and small G-protein (21). Focal adhesion proteins are integrin-rich microdomains, which transmit mechanical signals from the ECM to activate signaling pathways inside the cell and structurally link the cytoskeleton to the ECM (22). This result is consistent with the strong correlation between *EVA1B* and vimentin in glioma. Additionally, *EVA1B* was also associated with NF-kappa B signaling, the apoptotic signaling pathway, and TNF signaling pathway. These three signaling pathways were known to be associated with immune and inflammation processes.

Another important finding in this study is that *EVA1B* expression has a positive correlation with the abundance of infiltrating B cells, CD4^+^ T cells, CD8^+^ T cells, macrophages, and neutrophils in glioma. Furthermore, the immune cells were involved in the tumor immune network (23). For instance, Neeta Somalia et al. found that *LV305* could selectively induce *NY-ESO-1* expression of DCs, which further promotes tumor response in patients (24). The abundance of immune cells affects the outcomes of patients in head and neck squamous cell carcinoma (HNSCC) and lung adenocarcinoma (LUAD) (25, 26), which is in agreement with the results of this study. However, the role of immune cells in tumors is very complex. As studies have reported, on the one hand, DCs are professional antigen-presenting cells, which can capture and process antigen to present antigenic peptides on MHC Class I and Class II to activate CD8^+^ and CD4^+^ cells respectively (27). On the other hand, DCs could promote breast cancer bone metastasis *via* increasing Treg cells and reducing CD8^+^ cytotoxic T cells (28) and play a crucial role in cell proliferation, invasion, and intercellular communication (5, 29). At present, Treg cells are considered to be one of the main reasons for glioma producing an immunosuppressive microenvironment (30). Several studies have suggested that the proportion of Treg cells increases with the malignant degree of glioma (31).

Previous studies have mainly focused on the correlations between new antigens and survival by genomics (32). A comprehensive understanding of the immune infiltration status of tumor patients is of great significance for patients in choosing individual immunotherapy (33). In recent years, some immune-associated markers, such as *PD-L1*, tumor mutational burden (TMB), have been reported to become the indicators for immunotherapy response (34, 35). However, the dynamics of the immune microenvironment, other checkpoint molecules, infiltrating immune cells, and immune biomarkers may also be important for the response to immunotherapy (36). In recent years, the study of immune checkpoints has made a breakthrough in the field of tumor immunotherapy, and immune checkpoint blockade has also been approved for the treatment of melanoma and lung cancer (37). Although *PD-L1* expression could predict the immunotherapy response of some patients, PD-L1 expression is not enough to predict which patients should be treated with immunotherapy (38, 39). Therefore, an accurate precision biomarker is of great importance to individualized immunotherapy. Jing-Hua Pan et al. (40) recently found that *LAYN* overexpression is closely related to the abundance of immune cell infiltration in gastric and colon cancer, and may be involved in the regulation of tumor-associated macrophages, DCs, T cell exhaustion, and Tregs function. They concluded that overexpression of *LYAN* can be used as a molecular biomarker of immune infiltration and survival prognosis in patients with gastric or colon cancer. Similarly, we found that *EVA1B* was significantly correlated with immune checkpoint blocking molecules *PD-1, CTLA4, LAG3*, and *TIM3*, which suggested that *EVA1B* has the potential to act as a predictive biomarker for the effectors of immune checkpoint blockade in glioma. However, whether the same is true for other types of cancer remains to be studied.

There were limitations to the present study. Firstly, this study did not further verify the functional role of *EVA1B* in the immune microenvironment of glioma. This subject is very novel and worthy of further study. Secondly, although we found that *EVA1B* was mainly localized to cytoplasm and membrane in immunofluorescence images from HPA, the result should be verified by different cell lines. Thirdly, the present findings may be the basis for further studies to validate the outcomes.

In conclusion, we discovered for the first time that *EVA1B* was significantly upregulated in glioma, and increased with tumor grade. *EVA1B* overexpression was remarkably associated with malignant behavior and poorer prognosis in glioma patients. Another important finding is that the expression level of *EVA1B* correlates with the infiltration levels of immune cells including B cells, CD4^+^ T cells, CD8^+^ T cells, macrophages, and neutrophils. Thus, *EVA1B* is strongly correlated with the overall immune infiltration levels of glioma and could serve as an independent prognosis biomarker for glioma patients.

## Data Availability Statement

Publicly available datasets were analyzed in this study. This data can be found here: CGGA (http://cgga.org.cn/, Dataset ID: mRNAseq_325) and TCGA (http://cgga.org.cn/, Dataset ID: mRNAseq_325) database, and requests for further access to datasets can be directed to huayuanlu402@126.com.

## Ethics Statement

The studies involving human participants were reviewed and approved by Ethics Committee of Lishui People’s hospital. The ethics committee waived the requirement of written informed consent for participation.

## Author Contributions

JL and HW conceived the study. JL and SQ performed data analysis. SQ wrote the manuscript. HW revised the manuscript. All authors contributed to the article and approved the submitted version.

## Conflict of Interest

The authors declare that the research was conducted in the absence of any commercial or financial relationships that could be construed as a potential conflict of interest.

## Abbreviations

CNS, central nervous system; OS, overall survival; GBM, glioblastoma; IHC, immunohistochemistry. EMT, epithelial-mesenchymal transition; TMB, tumor mutational burden; HNSCC, head, and neck squamous cell carcinoma; LUAD, lung adenocarcinoma.
