# Supplementary material for: EVA1B to Evaluate the Tumor Immune Microenvironment and Clinical Prognosis in Glioma
Source: Front Immunol. 2021 Apr 6;12:648416. doi: 10.3389/fimmu.2021.648416 (PMC8056259; doi:10.3389/fimmu.2021.648416)
Supplement: Supplementary file 1 [file DataSheet_1.docx]

***Supplementary Material***

Catalogue

[1. **Supplementary Tables** 2](#_Toc64575587)

[1.1.Supplemental Table 1 2](#_Toc64575588)

[1.2 Supplementary Table 2 3](#_Toc64575589)

[1.3 Supplementary Table 3 4](#_Toc64575590)

[2. **Supplementary Figures** 5](#_Toc64575591)

[2.1 Supplementary Figure 1 5](#_Toc64575592)

[2.2 Supplementary Figure 2 6](#_Toc64575593)

[2.3 Supplementary Figure 3 7](#_Toc64575594)

[2.4 Supplementary Figure 4 8](#_Toc64575595)

[2.5 Supplementary Figure 5 9](#_Toc64575596)

[2.6 Supplementary Figure 6 10](#_Toc64575597)

1. **Supplementary Tables**
   1. Supplemental Table 1

Supplementary Table 1. Clinicopathological characteristics of patients in CGGA cohort and TCGA cohort.

| **Characteristics** | **CGGA cohort (n=325)** | **TCGA cohort (n=609)** |
| --- | --- | --- |
| Age(mean±SD) | 42.9±12.0 | 47.3±15.3 |
| Sex (male), n(%) | 203(62.5) | 354(58.1) |
| WHO grade, n(%) |  |  |
| WHOII | 103(31.7) | 216(35.5) |
| WHOIII | 79(24.3) | 241(39.6) |
| WHOIV | 139(42.8) | 152(25.0) |
| Histopathology |  |  |
| O | 26(8.0) | 117（19.2） |
| OA | 38(11.7) | 44（7.2） |
| A | 39(12.0) | 55（9.0） |
| AO | 12(3.7) | 87（14.3） |
| AOA | 39(12.0) | 40（6.6） |
| AA | 28(8.6) | 114（18.7） |
| GBM | 139(42.8) | 152（25.0） |
| IDH status, n(%) |  |  |
| mutation | 175(53.8) | 378(62.1) |
| Wildtype | 149(45.8) | 225(36.9) |
| 1p/19q status, n(%) |  |  |
| Codeletion | 67(20.6) | 151(24.8) |
| Non-codeletion | 250(76.9) | 453(74.4) |
| MGMTp methylation, n(%) |  |  |
| Yes | 157(48.3) |  |
| No | 149(45.8) |  |
| Radiotherapy, n(%) |  |  |
| Yes | 178(54.8) |  |
| No | 51(15.7) |  |
| Chemotherapy, n(%) |  |  |
| Yes | 178(54.8) |  |
| No | 124(38.2) |  |
| Recurrence, n(%) |  |  |
| Yes | 62(19.1) |  |
| No | 259(79.7) |  |

O: oligodendroglioma; OA: oligoastrocytoma; A: astrocytoma; AO: anaplastic oligodendroglioma; AOA: anaplastic oligoastrocytoma; AA: anaplastic astrocytoma; GBM: glioblastoma; MGMTp: MGMT promoter.

## 1.2 Supplementary Table 2

Supplementary Table 2. Spearman analysis of the correlation between EVA1B and clinicopathological variables (CGGA).

| Variables | EVA1B expression level | |
| --- | --- | --- |
|  | Spearman Correlation | *P*-value |
| Age | 0.25 | <0.001 |
| Sex | 0.02 | 0.679 |
| WHO grade | 0.52 | <0.001 |
| Histopathology | 0.52 | <0.001 |
| IDH status | 0.50 | <0.001 |
| 1p/19q status | 0.33 | <0.001 |
| MGMTp methylation | 0.09 | 0.109 |
| Radiotherapy | -0.04 | 0.460 |
| Chemotherapy | 0.14 | 0.019 |
| Recurrence | 0.04 | 0.445 |

MGMTp: MGMT promoter.

## 1.3 Supplementary Table 3

Supplementary Table 3. The correlation between the expression of EVA1B and clinicopathological factors (TCGA).

| **Feature** | **EVA1B expression level** | | ***P*-value** |
| --- | --- | --- | --- |
|  | **Low expression** | **High expression** |  |
| Age |  |  |  |
| ≥40 | 150 | 235 | <0.001 |
| <40 | 154 | 70 |  |
| Sex |  |  |  |
| Male | 168 | 186 | 0.153 |
| Female | 136 | 119 |  |
| WHO grade |  |  |  |
| WHO II | 166 | 50 | <0.001 |
| WHO III | 136 | 105 |  |
| WHO IV | 2 | 150 |  |
| Histopathology |  |  |  |
| O | 98 | 19 | <0.001 |
| OA | 31 | 13 |  |
| A | 37 | 18 |  |
| AO | 60 | 27 |  |
| AOA | 22 | 18 |  |
| AA | 54 | 60 |  |
| GBM | 2 | 150 |  |
| IDH |  |  |  |
| Mutation | 275 | 103 | <0.001 |
| Wildtype | 27 | 198 |  |
| 1p/19q |  |  |  |
| Codel | 122 | 29 | <0.001 |
| Non-codel | 182 | 271 |  |

O: oligodendroglioma; OA: oligoastrocytoma; A: astrocytoma; AO: anaplastic oligodendroglioma; AOA: anaplastic oligoastrocytoma; AA: anaplastic astrocytoma; GBM: glioblastoma.

1. **Supplementary Figures**
   1. Supplementary Figure 1


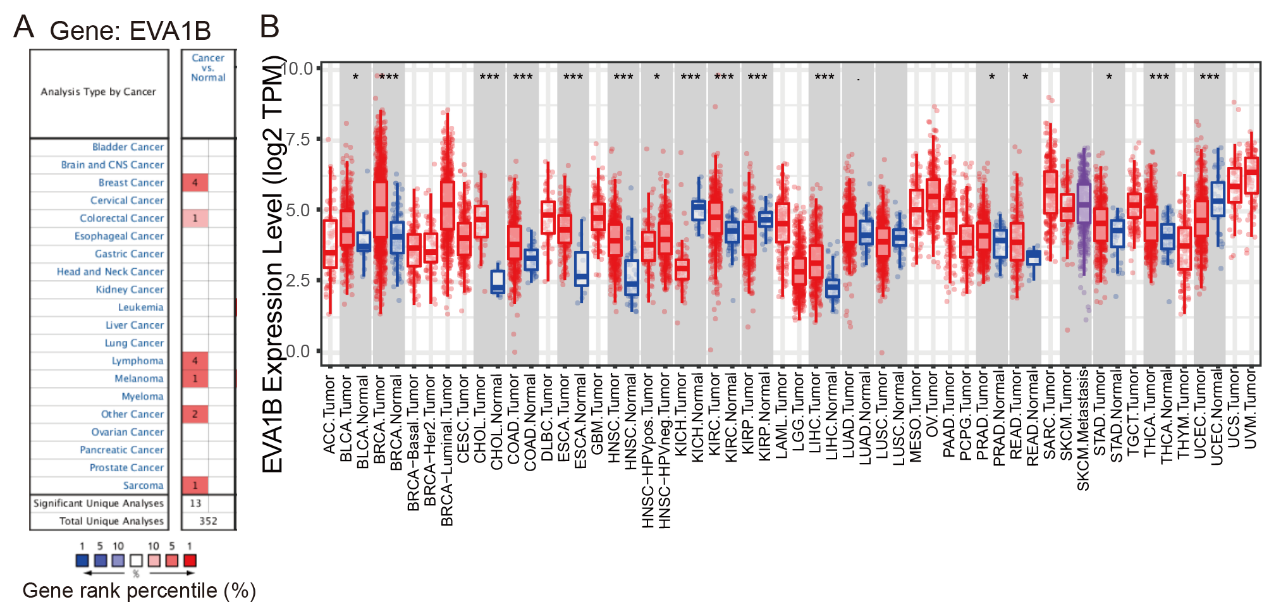


**Supplementary FIGURE 1 ⎜** Pan-cancer analysis of EVA1B expression levels in different databases. **(A)** Comparison outcomes from datasets of Oncomine. Red indicates high expression, blue indicates low expression. **(B)** EVA1B expression in Pan-cancer analysis using the TIMER tool. Red indicates tumor tissues and blue indicates normal tissues.

- 1. Supplementary Figure 2

**
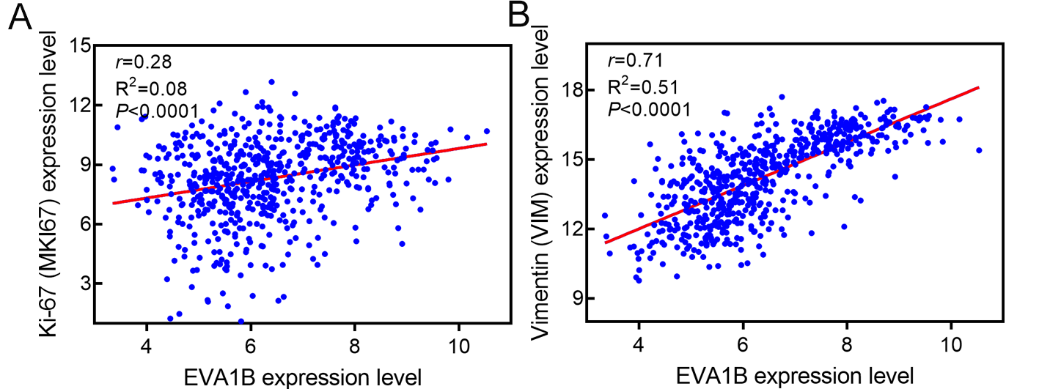
**

**Supplementary FIGURE 2** ⎜ The validation of the correlation between EVA1B expression and other indexes (TCGA cohort, n=609). (**A**) EVA1B expression is very weakly correlated with Ki-67 expression. (**B**) EVA1B expression is strongly correlated with vimentin expression.

- 1. **Supplementary Figure 3**


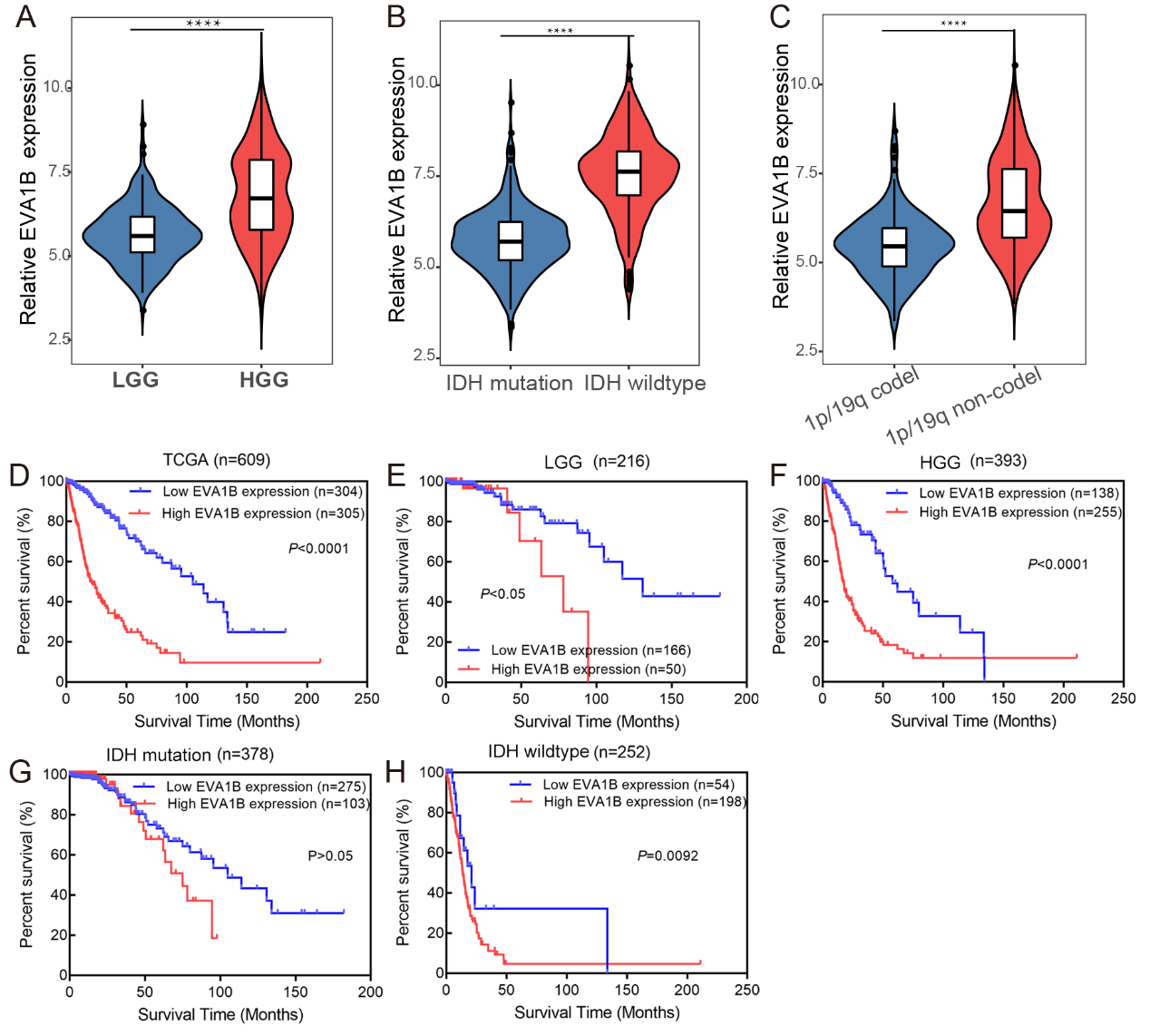


**Supplementary FIGURE 3 ⎜** Validation of overexpression and poor prognosis for glioma patients in the glioma cohort from TCGA (n=609). **(A-C)** The *EVA1B* expression was also significantly associated with WHO grade, IDH wildtype, and 1P/19q non-codeletion in the TCGA cohort. **(D-H)** Patients with high *EVA1B* expression showed significantly poorer prognosis than those with low *EVA1B* expression in the TCGA cohort and the subgroups stratified by WHO grade and IDH status, except for the IDH mutation subgroup.

## 2.4 Supplementary Figure 4


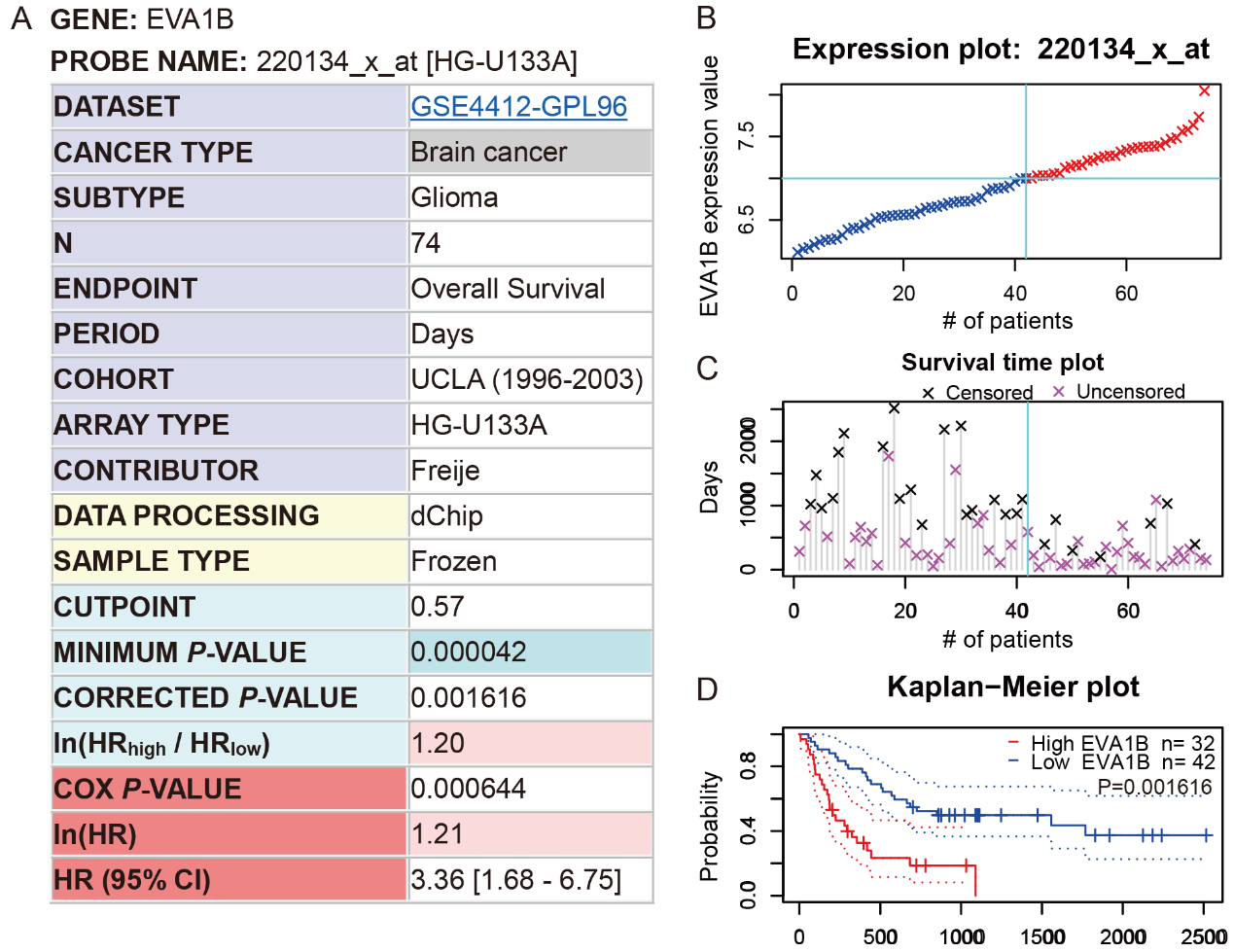


**Supplementary FIGURE 4 ⎜** Validation of the prognostic value of *EVA1B* overexpression in the glioma cohort from GEO (GSE4412-GPL96 dataset, n=74). **(A)** Screenshot of the PrognoScan report with final corrected P-value at 0.001616. **(B)** Screenshot of PrognoScan's expression gradient-based log rank p-values plot. The blue vertical line indicates where the minimal p-value is that was used for the corrected P-value. **(C)** Comparison of survival time and survival status between high and low *EVA1B* subgroups. **(D)** Kaplan-Meier plot between high *EVA1B* subgroup and low *EVA1B* subgroup.

## 2.5 Supplementary Figure 5


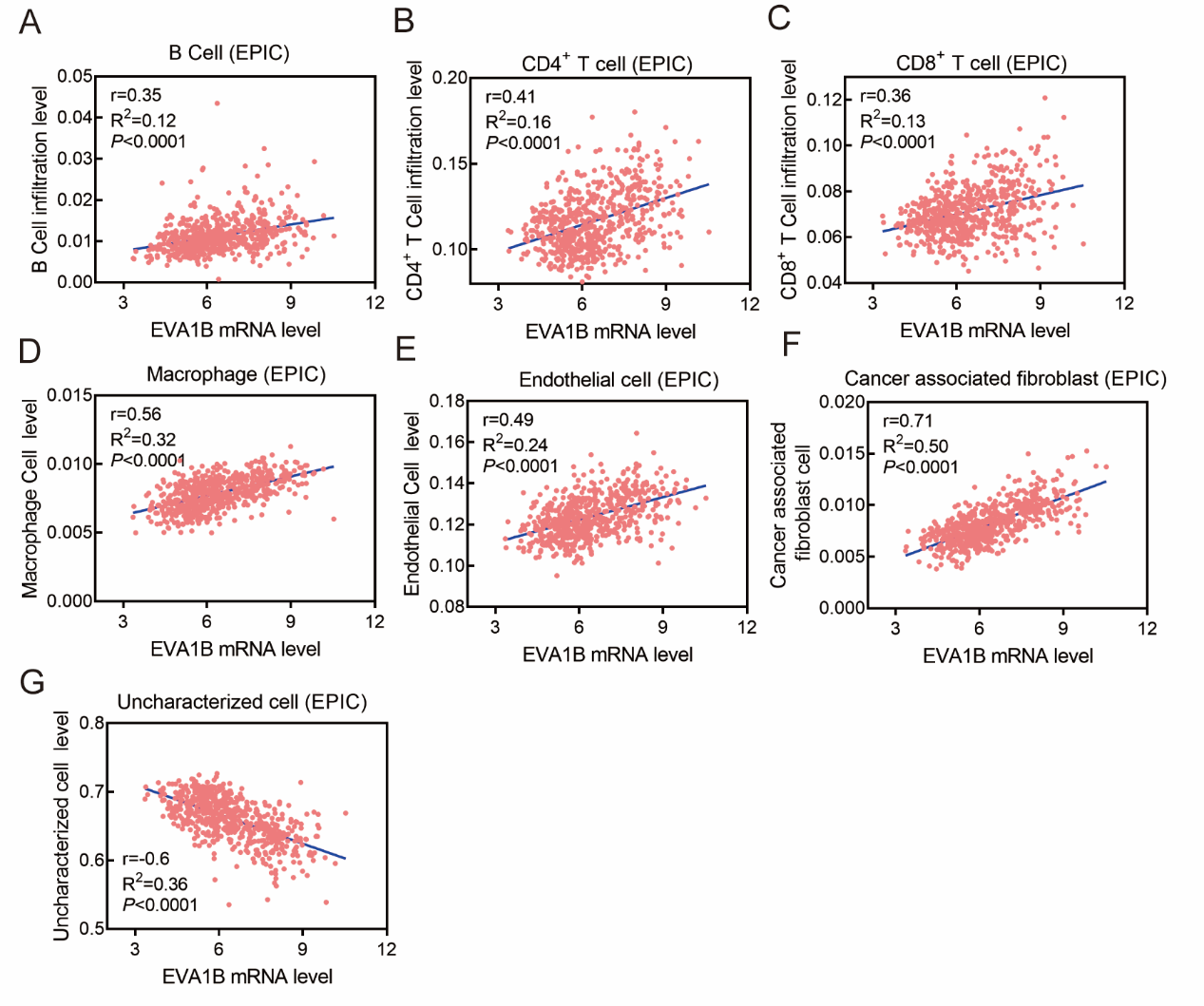


**Supplementary FIGURE 5 ⎜** The association between EVA1B expression and immune infiltration level based on EPIC algorithm. (**A-F**) EVA1B expression is correlated with infiltration levels of B cells, CD4^+^ T cells, CD8^+^ T cells, macrophages, endothelial cells and cancer associated fibroblast. (**G**) There was a strong negative correlation between EVA1B and uncharacterized cells.

## 2.6 Supplementary Figure 6


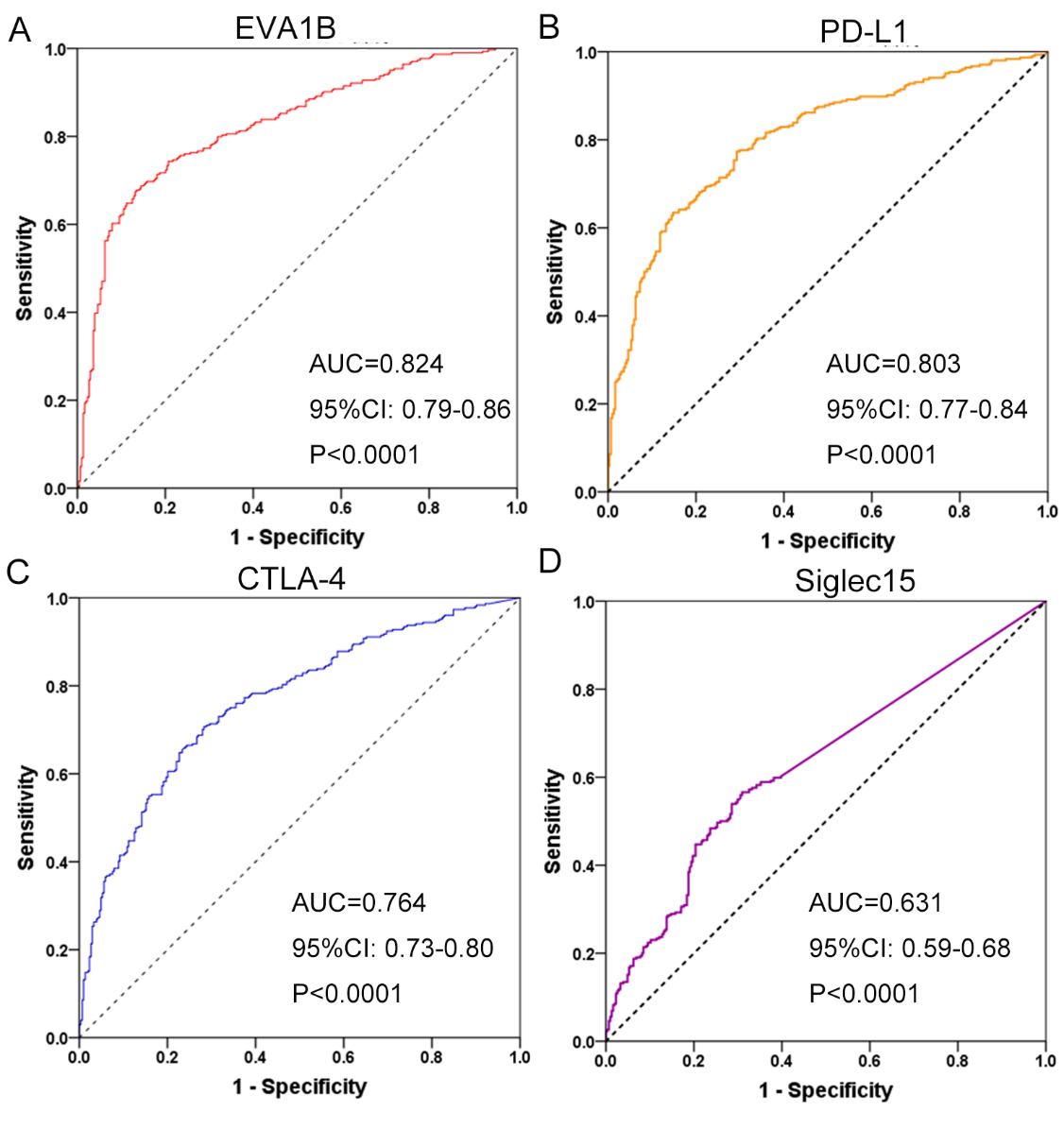


**Supplementary FIGURE 6 ⎜** Comparison of predictive power for high glioma immune-infiltration between *EVA1B* and common markers. (**A-D**) ROC curves showed that, compared with PD-L1, CTLA-4, and Siglec15, EVA1B presented a higher predictive power for assessing high immune-infiltration levels in glioma.
